# Supplementary material for: Preliminary demonstration of a persistent Josephson phase-slip memory cell with topological protection
Source: Nat Commun. 2021 Aug 31;12:5200. doi: 10.1038/s41467-021-25209-y (PMC8408200; doi:10.1038/s41467-021-25209-y)
Supplement: Supplementary file 1 — Supplementary Information [file 41467_2021_25209_MOESM1_ESM.pdf]

Supplementary Information: Preliminary Demonstration of a Persistent Josephson Phase-Slip Memory Cell with Topological Protection

Nadia Ligato,[1,*] Elia Strambini,[1,†] Federico Paolucci,[2,1,‡] and Francesco Giazotto[1,§]

[1] *NEST, Istituto Nanoscienze-CNR and Scuola Normale Superiore, I-56127 Pisa, Italy*[¶]

[2] *INFN Sezione di Pisa, Largo Bruno Pontecorvo, 3, I-56127 Pisa, Italy*

Note 1. Hysteresis in the current vs voltage characteristics

To test the PSM transport properties and highlight the hysteresis in its magneto-resistance, we electrically characterized the device at $T = 25$ mK. Figure S1 shows the current vs voltage characteristics ($I(V)$) of a typical PSM measured at $\Phi = 0.45\Phi_0$ (blue curve), $\Phi = 0.5\Phi_0$ (red curve) and $\Phi = 0.54\Phi_0$ (green curve) for positive (left) and negative (right) sweeps of the magnetic flux. The tunnel $I(V)$ characteristics reveal a magnetic-flux-induced modulation of the superconducting gap of the Al nanowire (Δ_w), and hysteric behavior with Φ, showing a maximum reduction of Δ_w at $\Phi = 0.54\Phi_0$ and $\Phi = 0.45\Phi_0$ for the forward and backward traces, respectively.

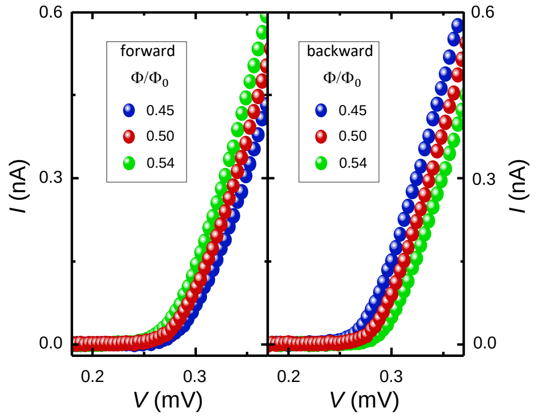

FIG. S1: **Current (I) vs Voltage (V) curves measured at different values of magnetic flux (Φ).** Left panel: $I(V)$ recorded while increasing Φ (forward). Right panel: $I(V)$ recorded while decreasing Φ (backward). All data were recorded at $T = 25$ mK.

*Electronic address: nadia.ligato@nano.cnr.it
†Electronic address: e.strambini@sns.it
‡Electronic address: federico.paolucci@nano.cnr.it
§Electronic address: francesco.giazotto@sns.it
¶Present address: TeCIP Institute, Scuola Superiore Sant'Anna, Pisa, IT

Note 2. PSM in continuous read-out configuration for several applied voltage biases

In order to find the optimal operating parameters for the PSM, we performed the writing/erasing operations in the continuous read-mode, i.e., when a bias voltage (V) is permanently applied, varying the values of V from 200 μV to 600 μV, as shown in Fig. S2 at $T = 25$ mK. The bias flux is set just above the crossing-point of the hysteresis, namely at $0.54\Phi_0$. The memory can be written or erased by applying a flux pulse down to $\Phi_W = 0.33\Phi_0$ or up to $\Phi_E = 0.75\Phi_0$, respectively.

The PSM shows the typical behavior of a memory cell with distinct current values for [0] and [1] states for $V \geq 300;\mu$V. By increasing V the visibility between the two stored states increases until saturating for the largest biases. This trend is due to local overheating in the weak link induced by the quasiparticle current flowing through the probing junction, which increases ξ_w thereby deviating the CPR towards the single-valued non-hysteretical form. Finally, we stress that the memory works properly for many bias cycles, confirming the *endurance* of the PSM cell.

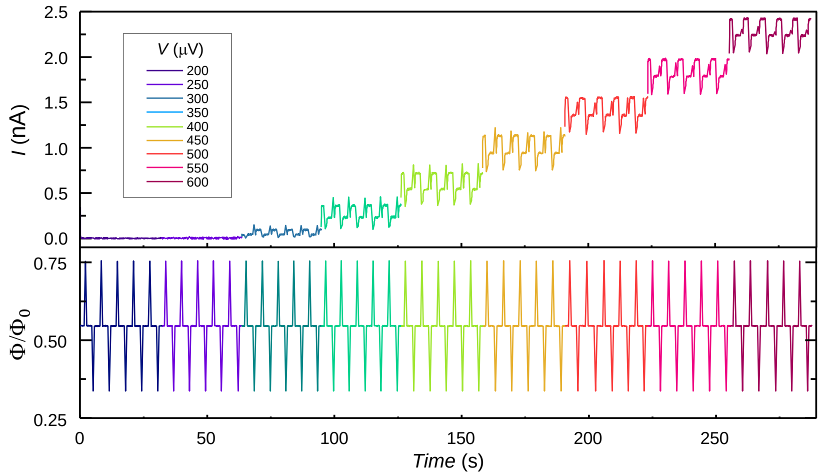

FIG. S2: **Writing/erasing operations in the continuous read-out mode performed for different values of permanently applied voltage bias (V).** Bottom: evolution of the magnetic flux composed by a bias at ($\Phi_R = 0.54\Phi_0$) and pulses to write ($\Phi_W = 0.33\Phi_0$) and erase ($\Phi_E = 0.75\Phi_0$) the memory state. Top: evolution of the read-out current (I) measured for different values of voltage bias (V). The higher (lower) current value $I_{[1]}$ ($I_{[0]}$) acquired for a fixed V corresponds to logic state [1] ([0]). $I_{[1]}$ and $I_{[0]}$ are negligible for $V < 300\mu$V, therefore the PSM cannot be biased in that voltage range. All data were acquired at $T = 25$ mK.

Note 3. Non-volatility of the PSM measured at different voltage biases

Here, we study the *non-volatility* of the PSM. Since the quiescent magnetic flux could be provided by a metallic ferromagnet buried in the isolating substrate or by a ferromagnetic insulator dielectric, the only power source relevant for the PSM is the voltage bias (V). Figure S3 shows the non-volatility of the device measured for several values of V. Temporarily removing the voltage bias has no effect on the stored data. In fact, the reading voltage is set to V only during the readout operation. In addition, the writing, erasing and reading operations have been performed several times without any sizeable outcome change.

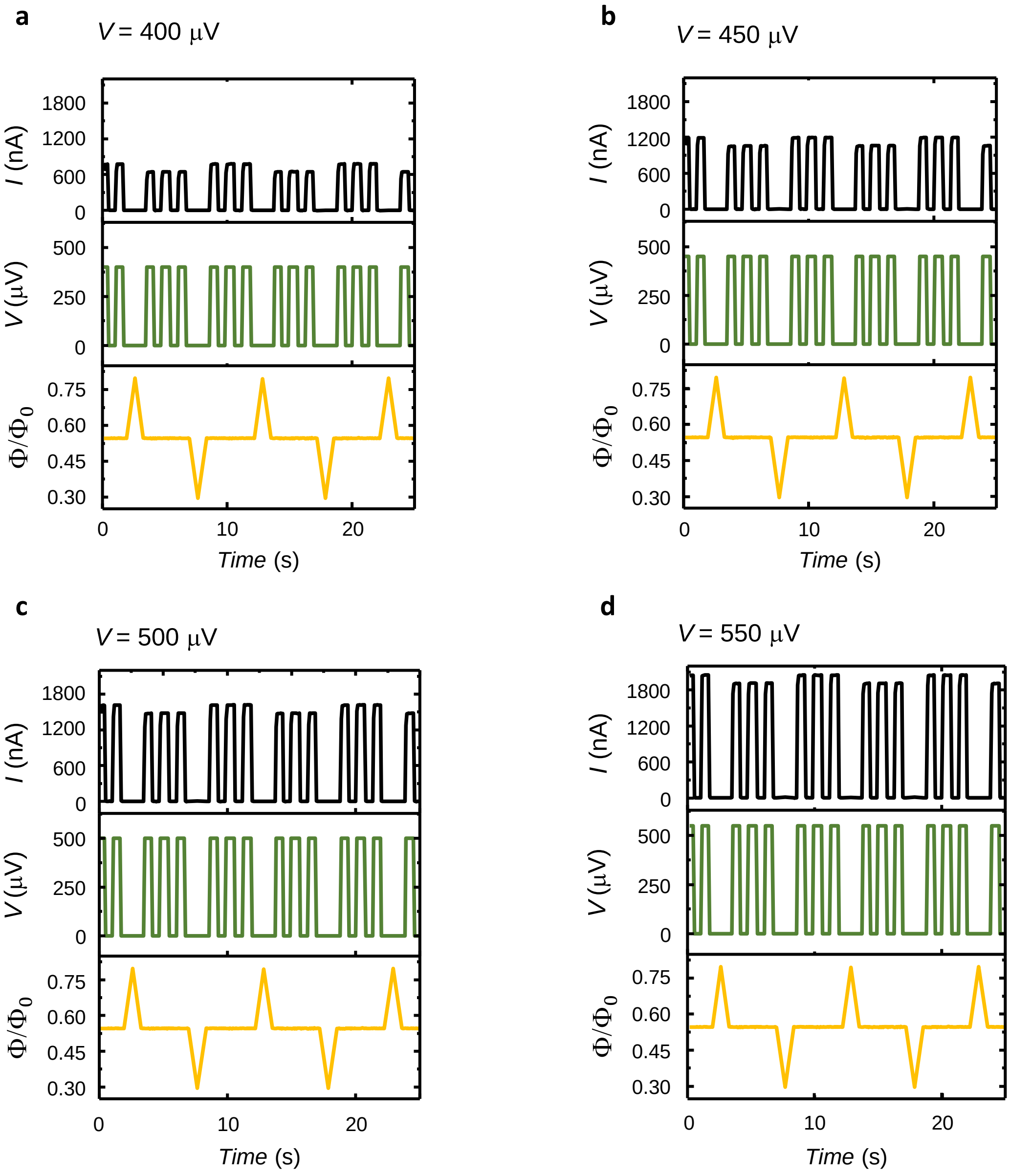

FIG. S3: **Writing/erasing operations performed for different values of a pulsed readout voltage bias (V).** V ranges form: 400 μV (**a**), 450 μV (**b**), 500 μV (**c**) and 550 μV (**d**). For each panel is shown the temporal evolution of the magnetic flux applied to the PSM (bottom) together with the bias voltage pulses (center) and the resulting tunnel current (top) used for the state readout. The flux was biased at $\Phi_B = 0.54\Phi_0$ while flux pulses of $\Phi_W = 0.33\ \Phi_0$ and $\Phi_E = 0.75\ \Phi_0$ were used for write and erase operations, respectively. The permanency of the memory state before and after the application of the readout bias demonstrate the persistency and non-volatility of the PSM. Note that the PSM works properly during the operation of the device for a wide range of applied voltage biases. All measurements were performed at $T = 25$ mK.

Note 4. PSM operation at the degenerate flux $\Phi_B = 0.5\Phi_0$

This section is devoted to the study of the properties of the PSM when operated at the degenerate flux $\Phi_B = 0.5\Phi_0$, where the two current branches are expected to show the same value. To this end, we bias the device with a constant voltage (V) while a sinusoidal oscillation of the magnetic flux is superimposed on $\Phi_B = 0.5\Phi_0$. Figure S4 confirms that the average values of $I_{[0]}$ and $I_{[1]}$ are indistinguishable. Conversely, the readout current oscillates in reversed phase depending on the stored state. Therefore, the information of the memory state is stored in the phase of the current signal, thus enabling the design of a phase-dependent readout.

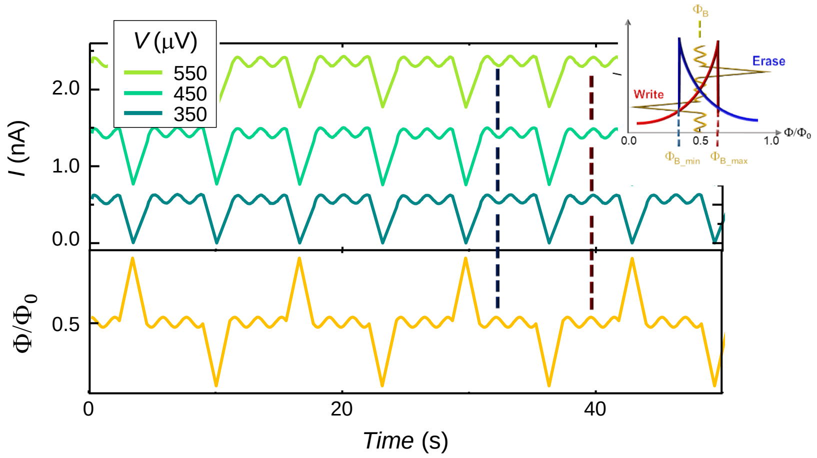

FIG. S4: **Writing/erasing operations performed at the degenerate point $\Phi_B = 0.5\Phi_0$.** Bottom: Temporal evolution of the total flux applied to the PSM and composed by a bias $\Phi_B = 0.5\Phi_0$ superimposed to a low frequency sinusoidal fluctuation with amplitude $\Phi_{AC} = \pm 0.04\Phi_0$. Write ($\Phi_W = 0.30\Phi_0$) and erase ($\Phi_E = 0.80\Phi_0$) pulses are applied to switch the memory state. Top: read-out current acquired at different voltage bias (V). Notice that at the degeneracy point the average values of $I_{[0]}$ and $I_{[1]}$ are indistinguishable while information of the memory state is stored in the phase of the signal. $I_{[0]}$ and $I_{[1]}$ show a 180° phase shift.Inset: scheme of operation in the $I(\Phi)$ diagram. All measurements were performed at $T = 25$ mK.

Note 5. Phase-dependent read-out scheme

The phase-dependent read-out can be realized by means of the experimental setup shown in Fig. S5a. The total magnetic flux is the sum of a constant component $\Phi_{\mathrm{B}} = 0.5\Phi_0$ (due to the current I_B flowing in the superconducting magnet) and a small sinusoidal component Φ_{AC} (due to the current I_{AC}). The PSM is biased with a constant voltage V. The phase of the output current (ϕ) is measured with a lock-in amplifier with respect to the I_{AC} oscillations. The results are summarized in Fig. S5b. The readout current for state [1] oscillates in phase with the magnetic flux oscillations, while state [0] shows a counter phase (180°) fluctuation. We note that the device unequivocally shows the typical behavior of a memory cell upon many erasing/writing cycles even for the phase-dependent readout.

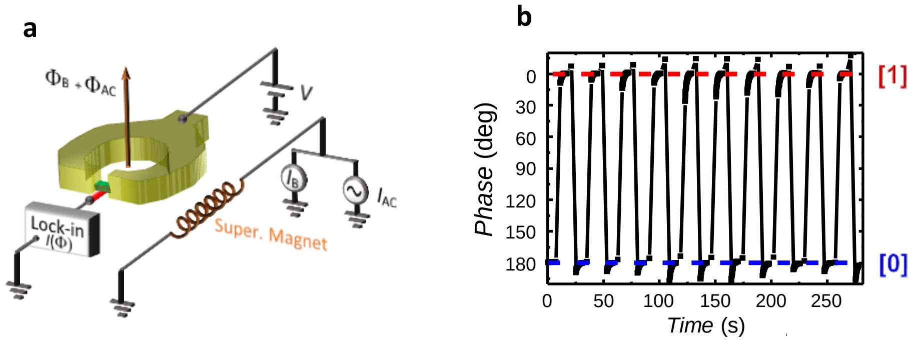

FIG. S5: **Read-out of the PSM by lock-in phase measurements a** Scheme of the PSM, where the Al ring (yellow), the Al nanowire (green) and the normal metal tunnel probe (red) are presented together with the complete electrical set-up. The PSM is biased with a DC constant voltage V= 400 μV. The magnetic flux piercing the superconducting ring is $\Phi_B + \Phi_{AC}$, where $\Phi_B = 0.5\Phi_0$ is the flux corresponding to the crossing point of the two current branches and Φ_{AC} is a small sinusoidal component superimpose with a lock-in source. The variations of the read-out current with the flux oscillations are recorded with standard lock-in technique. **b** Time dependence of the phase of the readout current while writing and erasing operations are performed. $I_{[1]}$ oscillates in phase with the magnetic flux (phase=0), while $I_{[0]}$ has the opposite dependence (phase=180°) allowing the acquisition of the two distinguishable signals of phase. All data were recorded at $T = 25$ mK.
